# Optimal Use of Novel Immunotherapeutics in B-Cell Precursor ALL

**DOI:** 10.3390/cancers15041349

**Published:** 2023-02-20

**Authors:** Federico Lussana, Gianluca Cavallaro, Pantaleo De Simone, Alessandro Rambaldi

**Affiliations:** 1 Department of Oncology and Hematology, University of Milan, 20122 Milan, Italy; 2 Azienda Socio Sanitaria Territoriale Papa Giovanni XXIII, 24127 Bergamo, Italy

**Keywords:** B-cell precursor acute lymphoblastic leukemia, relapsed/refractory, blinatumomab, inotuzumab, CAR-T cells

## Abstract

**Simple Summary:**

In this review, we discussed the impact of novel immune therapies on the treatment of child and adult acute lymphoblastic leukemia (ALL). Based on the promising results achieved in the relapsed/refractory (R/R) setting, several trials are currently evaluating the clinical benefit of incorporating these new drugs into frontline treatments. The results emerging from the most recent studies are opening new avenues to further the significant improvements in the clinical outcome of this disease.

**Abstract:**

Novel immune therapies are currently being used for patients with R/R ALL based on their ability to induce not only hematologic but also molecular remission. Despite promising results, specific clinical conditions, such as high tumor burden or extra medullary relapse, are still associated with a remarkably poor clinical outcome. Therefore, how to optimize the choice and the timing of such new treatments within different clinical settings remains a matter of debate. In addition, with the aim of increasing the rate and depth of molecular remission, clinical studies are currently evaluating the combination of these immunotherapies with chemotherapy in the contest of frontline treatment. The preliminary data suggest that this approach may increase the cure rate and perhaps reduce the use of allogeneic stem cell transplantation (alloHSCT) in first remission. In Ph-positive ALL, reproducible results are showing that frontline treatment programs, based on the combination of tyrosine kinase inhibitors and immunotherapy, can achieve unprecedented rates of hematologic and molecular remission as well as a long-term cure, even in the absence of chemotherapy and alloHSCT. The results from these studies have led to the development of potentially curative treatment modalities, even for older ALL patients who cannot be treated with conventional intensive chemotherapy. The present review examined the evidence for an appropriate use of the new immunotherapies in ALL patients and provided some appraisal of the current and future possible uses of these drugs for achieving further therapeutic improvement in the treatment of this disease.

## 1. Introduction

Acute lymphoblastic leukemia (ALL) is a hematologic malignancy that originates from the proliferation of a single B- or T-lymphocyte progenitor, which proliferates and accumulates in the blood; the bone marrow; and, possibly, the extramedullary sites. This disease represents the most frequent cancer in the pediatric population, especially in infants aged 1–4 years, with a subsequent decrease in the overall incidence which reaches its lowest point in young adults aged 25–45 years. The majority of ALL is of B-cell origin. Remarkable advances have been achieved in the treatment of ALL thanks to the optimal use of intensive chemotherapy, the implementation of minimal residual disease monitoring, and better supportive care, allowing most pediatric and adult patients to achieve a complete hematologic remission (CR). Despite these significant advances in treatment, less than 50% of adult patients survive 5 years or more, and about 15–20% of children suffer a relapse. Relapsed or refractory (R/R) ALL is still a major concern, with a dismal prognosis in both children and adults and with an overall survival of 3 to 12 months depending on the duration of the first remission and the number of lines of salvage therapy. In the last few years, the advent of novel targeted immunotherapies, specifically blinatumomab, inotuzumab, and chimeric antigen receptor T cells (CAR-T cells), has changed the therapeutic landscape of R/R B-cell precursor ALL (BCP-ALL). These novel immunotherapies showed impressive results in terms of response rates and a greater likelihood of proceeding to allogeneic stem cell transplantation (alloHSCT) consolidation, thus significantly changing the perspectives of this very unfavorable condition. Based on the promising results in the R/R setting, immunotherapies are currently under investigation in the early phases of the disease. Moreover, these targeted immunotherapies are usually well tolerated, so the associated clinical benefit is not limited to younger and fit patients but also to older and frail patients. The main objectives of this review were to focus on how to choose among these novel agents according to specific conditions and to highlight the important challenges to be addressed in the coming years.

## 2. Immunotherapies

### 2.1. Blinatumomab

Bispecific T-cell engagers (BiTE) are a class of bispecific antibodies designed for cancer immunotherapy [1]. Among these, blinatumomab is a bispecific T-cell engager with a dual affinity for CD3 and CD19 that brings effector T lymphocytes and target B cells into close proximity, allowing T cells to recognize and exert their cytotoxic activity against CD19-positive B cells. Blinatumomab has been approved by the US Food and Drug Administration for the treatment of pediatric and adult patients with R/R BCP-ALL as well as for the treatment of those with measurable residual disease (MRD). Based on the results of the phase II studies on R/R adult ALL [2,3], the current treatment schedule is based on a ramp-up with 9 mcg daily over the first week followed by 28 mcg daily for 28 days in each subsequent cycle. The efficacy and safety of blinatumomab for the treatment of adult patients with R/R Ph-negative B-cell precursor ALL have been demonstrated through the pivotal, phase III TOWER study (ClinicalTrials.gov identifier: NCT02013167) that randomized (2:1 ratio) 405 patients to either blinatumomab monotherapy or multiagent chemotherapy [4]. Compared with the chemotherapy arm, the blinatumomab-treated patients achieved a significantly better complete remission (CR, 34% versus 16%), event-free survival (EFS, 31% versus 12%), and median overall survival (OS, 7.7 months versus 4.4 months), no matter their age, prior to salvage treatment or prior to alloHSCT. Among the patients who achieved CR, 76% were MRD negative in the blinatumomab treatment group versus 48% in the chemotherapy group. These results have been confirmed in the pediatric setting. In a phase I/II dose-escalation/dose-expansion trial conducted on Ph-negative ALL patients with a refractory disease or a disease relapsed after at least two lines of therapy or alloHSCT, blinatumomab showed a complete remission rate of 39%, with MRD negativity in 52% of the responders. The median OS was 7.5 months [5]. In a phase III randomized study AALL1331 trial (ClinicalTrials.gov identifier: NCT02101853), the effect of postreinduction/consolidation with blinatumomab vs. chemotherapy was evaluated in patients < 30 years. Blinatumomab proved superior compared to chemotherapy, with a 2-year OS rate of 71% vs. 58%. The rate of MRD negativity was 75% for the blinatumomab group vs. 32% for the chemotherapy group (*p* < 0.001). Given the better response, the likelihood of proceeding alloHSCT was greater for the blinatumomab group (70% vs. 43%) [6]. In a parallel study, blinatumomab, compared with intensive multidrug chemotherapy, before alloHSCT was confirmed to have a clinical benefit, with an improved EFS (66% vs. 27%) and OS (85% vs. 70%) [7].

The efficacy of blinatumomab has also been tested as a single agent in patients with R/R Ph+ ALL (ALCANTARA; ClinicalTrials.gov identifier: NCT02000427), showing a response rate of 36% associated with a rate of complete MRD response of 88%, which was close to that reported in R/R Ph-negative ALL. Among the responders, 44% proceeded to alloHSCT. After a median follow-up of 16.1 months, the median relapse-free survival (RFS) was 6.8 months, while the median OS was 9.0 months in the whole cohort and 19.8 months in the blinatumomab responders [8]. A propensity score analysis was also performed to compare the outcomes of the patients treated with blinatumomab in the AZCANTARA study with those of an external historical cohort of patients receiving standard chemotherapy. A higher rate of CR/CRh (36% vs. 25%) and a better OS with a hazard ratio of 0.81 (95%CI, 0.57 to 1.14) was seen after treatment with blinatumomab [9].

Based on the significant results obtained in the R/R setting, blinatumomab was also evaluated in patients with molecular evidence of MRD in their first or later CR. In a multicenter phase II study, which was conducted in 116 adult patients with ALL in hematologic CR but with a high MRD level (>10^−3^), blinatumomab induced an excellent 78% molecular response rate after the first cycle. The median OS was 36.5 months, and a prolonged survival was occasionally achieved without alloHSCT [10]. Interestingly, for the patients in first CR achieving MRD negativity, there was no evidence that the OS improved in patients who did or did not undergo transplantation. On the contrary, the outcomes of the patients receiving blinatumomab who were in their second or later CR and who did not proceed to HCT were inferior [10]. On the basis of these results, the US Food and Drug Administration (FDA) and the European Medicines Agency (EMA) granted the first approval for the treatment of MRD with blinatumomab. In a large real-world study, blinatumomab confirmed strong efficacy outcomes in adults with MRD+ B-cell precursor ALL, with 91% of Ph− and 59% of Ph+ patients achieving an MRD response within two cycles of blinatumomab. The overall MRD response did not differ by the CR state (CR1 or CR2) in both the Ph− and the Ph+ subgroups [11].

Finally, an escalation phase Ib study (NCT04521231) is investigating the safety and efficacy of subcutaneous blinatumomab for the treatment of adults with R/R BCP-ALL. Blinatumomab given subcutaneously does not only simplify its administration but also maximizes the pharmacokinetics and dynamics while decreasing the patients’ discomfort. Preliminary results are in keeping with this hypothesis [12].

To sum up, despite the high rate of responses leading to the approval of blinatumomab as a single agent in R/R ALL, the cure rate remains rather limited without subsequent consolidation with alloHSCT [13]. Based on these observations and on the optimal results of blinatumomab in the treatment of MRD positive patients, an anticipated use of this drug in frontline therapy seems to be the best way to optimize its clinical benefit as we discuss later in this review.

### 2.2. Inotuzumab Ozogamicin

Inotuzumab ozogamicin is an anti-CD22 antibody conjugated to calicheamicin. Its regular approval by the FDA and the EMA for R/R B-cell precursor ALL came from a phase III trial, the INOVATE trial (ClinicalTrials.gov identifier: NCT 01564784) [13]. Compared with the conventional chemotherapy arm, patients treated with inotuzumab achieved a significantly higher rate of CR and CRi (81% versus 29%) and had a significantly better OS (median of 7.7 versus 6.7 months) and PFS (5 versus 1.8 months). Among the patients who achieved CR, 78% were found to be MRD negative through flow cytometry in the inotuzumab treatment group versus 28% in the chemotherapy group. The duration of remission was in favor of inotuzumab as confirmed by a long-term follow-up reporting a 3-year rate of 20.3% versus 6.5% in the control group [14]. Interestingly, inotuzumab remained efficacious in patients with a high baseline disease burden, and this did not seem to negatively impact the safety profile of this drug [15]. The main inotuzumab toxicity causing concern was hepatoxicity, including a more frequent incidence of veno-occlusive disease (VOD) (13% vs. 1%). About 3% of cases occurred in patients receiving inotuzumab therapy alone, while most cases occurred after alloHSCT and with the use of a dual-alkylator conditioning regimen.

Nonetheless, the survival with the inotuzumab single agent remained suboptimal. For this reason, inotuzumab was combined with low-intensity chemotherapy based on 8 cycles of mini-hyper-CVD with or without blinatumomab in 96 patients with BCP-ALL in their first relapse (29 with blinatumomab and 67 without). The majority of the patients achieved CR or CRi (80% for the entire cohort and 91% for those in their first relapse). Among 75 evaluable patients, for MRD assessment through MFC, 62 patients (83%) achieved negative MRD, with higher rates of MRD negativity in those in their first relapse (89%). Forty-four patients (46%) received alloHSCT. The 3-year OS rate was 33% for the entire cohort and 42% for those in their first relapse, which was superior to that observed with either inotuzumab or blinatumomab single agents when using a propensity score analysis [16,17,18,19]. In the SWOG1312 phase I trial, inotuzumab was combined with reduced-intensity chemotherapy with encouraging results. The last reported data showed a composite rate of remission of 61% among 23 heavily pretreated patients. The treatment was well tolerated, with three patients experiencing hepatic VOD after their second transplantation or when receiving the higher dose of inotuzumab [20]. Recently, the single-arm phase II trial AALL1621 (ClinicalTrials.gov identifier: NCT02981628), conducted by the Children’s Oncology Group, showed that inotuzumab is effective and well tolerated in heavily pretreated children and adolescents (age of 1–21 years) with R/R CD22-positive BCP-ALL. VOD after hematopoietic stem-cell transplantation and prolonged cytopenias were notable toxicities, while partial CD22 expression and lower CD22 site density were associated with a lower likelihood of response [21].

In summary, inotuzumab is approved as a single agent for relapsed/refractory B-cell precursor ALL due to the high rate of response in terms of CR and MRD negativity, but its efficacy remains limited by a short duration of response. As mentioned later in the review, to improve the clinical benefit of inotuzumab, ongoing studies are exploring its use in frontline therapies or as a bridge to allogeneic transplants or CAR-T cell therapy. Finally, VOD is still a concern, but the hematologic community is progressively learning to prevent this serious complication by avoiding a dual-alkylator conditioning regimen and by increasing the time between the last dose of inotuzumab and the conditioning regimen.

### 2.3. Chimeric Antigen Receptor T Cells

Cellular immunotherapy with chimeric antigen receptor T-cell (CAR-T cell) anti-CD19 represents an attractive therapeutic option in the R/R setting. Different generations of CAR-T cells have been developed with different costimulatory domains (commonly 4–1 BB or CD28), which are relevant due to their expansion and persistence [22]. Tisagenlecleucel (Tisa-cel) is the first CAR-T cell medication approved for R/R ALL for those up to 25 years of age. In the first phase I/IIa study, in 30 patients, the CR rate was 90%, 88% of whom achieved MRD negativity [23]. The responses were quite durable from 2 to 24 months, with 68% CAR-T cell persistence and B-cell aplasia at 6 months. A subsequent larger study including 79 children and young adults showed a CR rate and concomitant MRD negativity of 82%. In the long-term follow-up, the 5-year EFS and OS rates were 42% (95%CI, 29–54) and 55% (95%CI, 43–66), respectively [24,25]. In a pediatric real-life analysis, Tisa-cel was confirmed to be an effective salvage treatment. It is worth remembering that a high disease burden, defined as bone marrow blasts ≥ 5%, was associated with a significantly worse OS (58% vs. 85%) [26].

Based on the excellent remission rates and the significant clinical benefit observed particularly in the responding patients who achieved MRD negativity, the COG cooperative group launched the first trial of the earlier use of CAR-T cell therapy in the setting of patients with MRD positivity. The AALL1721/Cassiopeia study (Clinical Trials.gov identifier: NCT03876769) was a phase II single-arm trial of tisagenlecleucel in children and young adults in CR1 with high-risk criteria and persistent MRD positivity (>0.01% through flow cytometry) at the end of chemotherapy. The primary end point of this study was the 5-year DFS, and the secondary end points included the overall survival, safety, the percentage of patients in remission without alloHSCT at 1 year, and the time to reach B-cell recovery.

In the adult patients, although the impressive rate of CR was confirmed, the long-term outcomes achieved with academic CAR-T cells were initially less significant, with a median EFS and OS of 6.1 months and 12.9 months [27]. The largest set of safety and efficacy data on Tisa-cel in the real-world setting included 255 children and young adult ALL patients. With a median follow-up of 13.4 months and a CR rate of 85.5%, the 12-month duration of response (DOR), EFS, and OS rates were 60.9%, 52.4%, and 77.2%, respectively [28].

More recently, the single-arm ZUMA-3 trial evaluated KTE-X19 CAR-T cell therapy in the largest population of adults with R/R B-cell precursor ALL to date. KTE-X19 allowed 39 patients (71%) to achieve CR or CRi, and 97% of these responders obtained MRD negativity. After a median follow-up of 16 months, the median overall survival was 18 months across all the treated patients. The safety profile of KTE-X19 was manageable and was without deaths due to cytokine release syndrome. These survival outcomes compare favorably with those achieved by blinatumomab and inotuzumab, leading the FDA to approve KTE-X19 as the first CAR-T cell product for adult ALL patients. [29]. Overall, however, a very recent systematic review of 16 studies involving 489 adults confirmed a very high early remission rate; however, the duration of response remains challenging [30], and the need for a subsequent allogeneic transplantation is often considered to be necessary [31,32,33,34].

A novel second-generation CD19 CAR-T cell therapy with a fast binding off rate for CD19 (AUTO1), designed with the aim to reduce toxicity and to improve persistence, was tested in a phase I study of 20 adult patients with R/R ALL. Interestingly, the study design planned a double infusion of CAR-T cells at different dosages according to the blast percentage. The preliminary results of this study were encouraging, with a high remission rate (85% had a MRD negative response) and an EFS and OS at 12 months of 48% and 64%, respectively. No patients experienced ≥ grade III CRS, and 15% experienced grade III neurotoxicity that resolved within 72 h with steroids [35]. To improve the clinical results and to shorten the time from cell collection to infusion into the patient, new laboratory approaches have been developed by our Chinese colleagues, such as the so-called FasT CAR-T (F-CAR-T) cell next-day manufacturing platform. The underlying idea is to freeze the cells a few hours after the viral transduction and to allow CAR-T cell expansion in vivo after the cells are thawed and infused back into the patient. Reducing the duration of the ex vivo culture should limit the differentiation of T cells, favoring the preservation of naïve and stem cell memory T cells in the final product. This is expected to be useful for obtaining a longer CAR-T cell persistence and, consequently, higher response rates and a longer duration of response. The preclinical studies and the first human clinical studies confirmed a superior in vivo CAR-T cell proliferation and expansion compared to conventional CAR-T cells, which showed a younger phenotype [36]. A similar clinical trial is also currently ongoing in the US and in Europe with a novel autologous CD19-targeting CAR-T cell manufactured using the T-Charge™ Platform (YTB323) for the treatment of R/R CD19-positive lymphoid neoplasms. The T-Charge™ Platform minimizes the ex vivo culture time and reduces the manufacturing process time to < 2 days. This novel platform also preserves naïve/TSCM cells in the final product, leading to a potentially higher potency and persistence [37] (Clinical Trials.gov identifier: NCT03960840).

**Figure 1:**
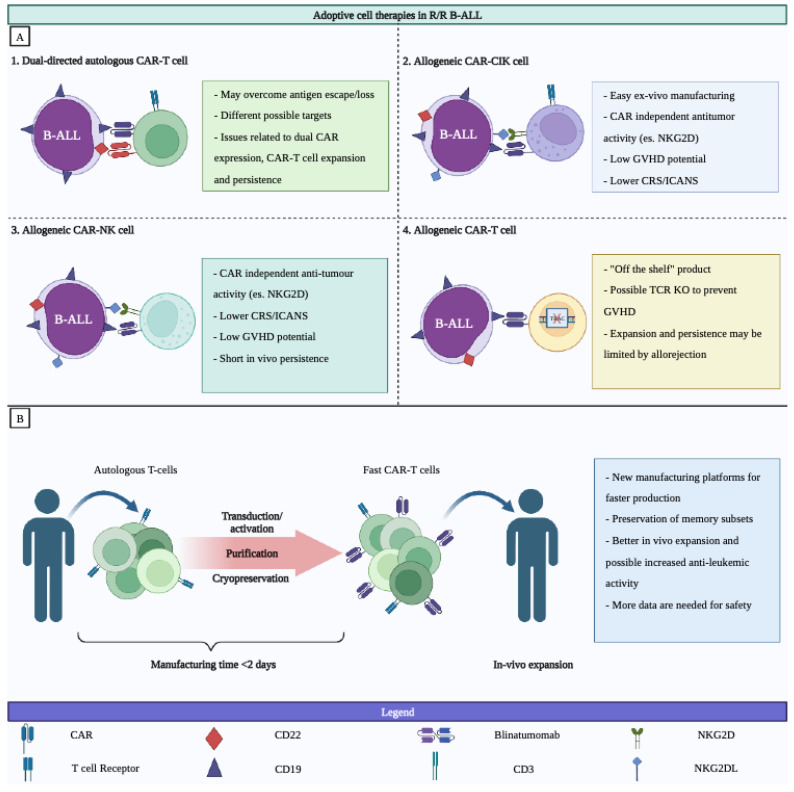
Novel approaches for exploration of immunotherapies in relapse/refractory BCP-ALL. Panel (**A**) new approaches with different adoptive cell therapies in relapse/refractory BCP-ALL Panel (**B**) key characteristics of the new manufacturing platforms for the fast production of CAR-T cells.

New approaches to overcoming the main obstacles of CAR-T cell therapy, such as the high costs and logistical complexity of the transduction viral process, and to allowing lymphodepleted patients to access CAR-T cell treatment are underway. In addition, the time of production may be clinically relevant, as some patients may face disease progression during this time, precluding the treatment itself. In this regard, to replace patient-derived CAR-T cells with ready-to-go cellular products, such as off-the-shelf allogeneic CAR-T cells, represents an ambitious goal of the ongoing research. However, the possibility of life-threatening graft-versus-host disease (GvHD) with an allogenic cell therapy product raises major concerns. Therefore, clinical research with immune cells other than “conventional” T cells is underway (Figure 1A,B).

ALL represents acute lymphoblastic leukemia, CAR represents chimeric antigen receptor, CIK represents cytokine-induced killer cells, CRS represents cytokine release syndrome, ICANS represents immune-effector-cell-associated neurotoxicity syndrome, NK represents natural killer, GVHD represents graft-versus-host disease, PD represents programmed death, T-reg represents regulatory T cells, and TRAC represents T-cell receptor alpha constant gene.

The figure was created with BioRender.com.

In a recent phase I and II clinical trial (NCT03389035), we examined the treatment feasibility of BCP-ALL patients who relapsed after alloHSCT with donor-derived anti-CD19 CAR-T cells (CARCIK-CD19) engineered with the sleeping beauty (SB) transposon. A complete response was achieved in 18 out of 27 patients (66.7%) and in 16 out of the 21 patients treated with the 2 highest doses (76.2%). A total of 14 (77.8%) of the overall responders and 13 of the responders at the highest doses (81.3%) were MRD negative. With a median follow up of 2.8 years, a significantly better EFS was observed in the patients treated with the highest doses (*p* = 0.0265). For the 21 patients treated at the 2 highest dose levels, the median EFS and OS were 4 and 12 months, respectively. The median DOR of the 16 patients in CR was 9.5 months, with a 6-month DOR of 54.4% (SE = 13.8). CRS occurred in nine patients (four patients with grade I CRS and five with grade II CRS), and immune-effector-cell-associated neurotoxicity (ICAN) (grade III) occurred in two patients at the two highest doses. Although 10 out of 27 had experienced GvHD after the previous alloHSCT, GvHD never occurred after treatment with CARCIK-CD19 [38,39]. In keeping with our results, a very recent phase I study of 25 patients treated with allogeneic CAR-T cells (UCART19), manufactured from healthy donor peripheral blood, confirmed the safety profile of allogeneic CAR-T cells, with a low risk of inducing GvHD (only 2 patients developed grade I acute cutaneous GvHD) [40].

Due to their ability to recognize tumor cells in a non-HLA (human leukocyte antigen)-restricted manner, other “nonconventional” allogeneic cells used in CAR-T cell technology are natural killer (NK) cells. Preliminary promising results have been reported in different tumors, such as multiple myeloma or neuroblastoma [41,42], but the observed short-term responses remain as the main concern for the use of this cell therapy. Liu and colleagues published a small study including 11 patients with CD19-positive lymphoid tumors. Eight patients (73%) achieved a hematologic response, and seven (four with lymphoma and three with CLL) had complete remission. The responses were rapid and were seen within 30 days after infusion at all dose levels. However, most patients had short-term responses, and five of eight patients needed post-remission therapy [43].

In conclusion, CAR-T cell therapy represents a potential curative treatment for R/R ALL, particularly when used for a low burden relapse or MRD positive disease. Given the complexity of this therapy, its possible early placing in ALL therapy remains a major open spot. Moreover, a subsequent allogeneic transplant is a matter of debate, and some biological parameters, such as B-cell aplasia and CAR-T cell persistence, could be helpful parameters in this difficult choice. A longer follow-up on the pivotal clinical trial will probably address some of these questions. Finally, there are many new platforms developed with the aim to make manufacturing easier and faster as well as to improve cell therapy efficacy. In this regard, it is worth noting that the time of production may be clinically relevant, as some patients may undergo clinical disease progression during this time, precluding the treatment itself.

A summary of the evidence leading to the regulatory approval of the immunotherapies (blinatumomab, inotuzumab, tisagenlecleucel, and brexucabtagene autoleucel) for R/R ALL is presented in Table 1.

## 3. Mechanisms of Resistance to Immunotherapies

Different mechanisms of tumor escape after treatment with novel immunotherapies have been described, and, in general, either the capacity of the leukemic clone to escape from the therapy-related immune selection or the inability of the immune system to generate an adequate response against leukemia can be referred to [44]. Some of these mechanisms of relapse are shared among the different therapies; others are treatment-specific and rely on the peculiar mechanism of action of each treatment.

The efficacy of blinatumomab depends on cytotoxic CD3+ T-cell engagement against CD19-expressing cells. The emergence of CD19-negative leukemic clones has been observed in about one-third of patients who relapsed after blinatumomab [45]. Multiple mechanisms can contribute to CD19 loss, such as alternative splicing, truncated CD19 variants, or a disrupted CD19 membrane export in the postendoplasmic reticulum compartment [46,47]. A myeloid lineage switch in patients with KMT2A(MLL) or ZNF384 rearrangements is also possible [48]. In addition, the possible mechanisms of resistance include the inhibition of blinatumomab-induced T-cell expansion due to exhausted T cells or the presence of an excessive number of regulatory T cells (T-regs) [49,50]. In a recent study using bulk tumor and single-cell sequencing on 44 R/R BCP-ALL patients, blinatumomab nonresponders displayed a T-cell repertoire with clonal expansion that was enriched in mucosal-associated invariant T cells (MAIT), unconventional T cells involved in nonpeptidic antigen recognition [47]. A threshold of T-regs of >10% in the peripheral blood has been proposed to identify patients with the highest likelihood to be resistant to blinatumomab [50]. The overexpression of PD1/PD-L1 can also be important [51]. Besides the quality of the T cell response, the tumor burden also affects the treatment outcomes. Patients with a bone marrow blast infiltration of >50% have a lower probability to achieve CR [4]. Thus, the optimal ratio between the CD3+ T cells and the target CD19+ cells is probably crucial. For such patients, a pretreatment phase is suggested to reduce the load of the disease, with the ultimate goal of also reducing adverse reactions, including cytokine release syndrome [52,53].

Similar to blinatumomab, downregulation or antigen loss is a common mechanism of tumor escape after anti-CD19 CAR-T cell therapy, with observed CD19-negative relapse rates of 10–25% in several clinical trials [23,24,27,54]. In a recently published paper, three main patterns of relapse were found. Among 420 CAR-T cell treated children and young patients, 51% were CD19-positive, 41% were CD19-negative, and 7% showed a lineage switch relapse. Notably, in a multivariable model, high preinfusion disease burden, prior blinatumomab nonresponse, older age, and a 4–1BB CAR-T cell construct were associated with an increased risk of CD19-negative relapses [55].

Among the different modalities of CD19 modulation, genetic and posttranscriptional aberrations have been well documented: frameshift code insertions; deletions in exons 2–5; and mRNA splice variants, in particular exon 2, are associated with the loss of either the transmembrane domain, with the consequent absence of surface antigen expression [56], or the extracellular epitope recognized by CAR-T cells [57]. Interestingly, the presence of CD19-negative subclones [58] or leukemic cells harboring exon 2 splicing variants [59] have also been observed in samples of patients with BCP-ALL at diagnosis, suggesting that the possible mechanisms of escape could be acquired even before exposure to the immunotherapies. Moreover, antigen masking due to accidental transfection and the consequential aberrant expression of the anti-CD19 CAR-T cells by BCP-ALL cells [60]; the transfer of CD19 from blast to T cells via a phenomenon known as trogocytosis [61]; or a phenomena of leukemia plasticity with phenotype conversion, in particular, in patients harboring KMT2A gene rearrangements [62,63,64] are other modalities by which leukemic cells can modulate antigen expression. Usually observed early after the infusion, relapses of BCP-ALL that maintain the expression of CD19 are associated with a failure of the CAR-T cells to expand and persists in vivo. In these cases, the mechanisms of resistance are related to some current limitations of CAR-T cell technology, in particular, the quality of the T cells selected for transfection (long-lasting memory vs. effector T cells), the ratio of the CD4+ and CD8+ T cells to be transfected, the choice of the costimulatory domain (4–1 BB vs. CD28), the affinity for CD19, and the antigenicity of the scFv [65].

The mechanisms of resistance to InO are less defined and are quite dissimilar from those described previously, giving a different targeted antigen and distinctive mode of action. In particular, a loss of CD22 expression by the leukemia at relapse is not frequent and has been described only in the case reports of both pediatric and adult BCP-ALL patients [66,67]. When analyzing the patients treated in the INOVATE trial, Kantarjian and coll. observed a reduction in CD22 expression from baseline in the subjects who relapsed after InO treatment, but no CD22-negative clones were detected [68]. Furthermore, the same group analyzed the bone marrow samples from the patients before treatment with InO and reported a reduction in CR and the DOR in those with lower blast CD22 expression compared with the patients with CD22 positivity ≥ 90% measured through flow cytometry [69]. Intriguingly, the patients with leukemic cells harboring KMT2A rearrangements were characterized at baseline by a lower expression of CD22 and, both in the subgroup analysis of the phase III trial and in the follow-up studies, these patients did not benefit from InO in terms of response and survival rates in comparison with the standard of care [13,69]. These data suggest that the efficacy of InO and the consequent risk of relapse are associated with the level of antigen expression and density on the leukemic blast, which influences the amount of the drug that is delivered into the cells. Finally, a recent work on pediatric patients with BCP-ALL showed that the specific aberrant splicing variants of the CD22 gene were associated with antigen downregulation and an acquired resistance to InO [70].

**Figure 2:**
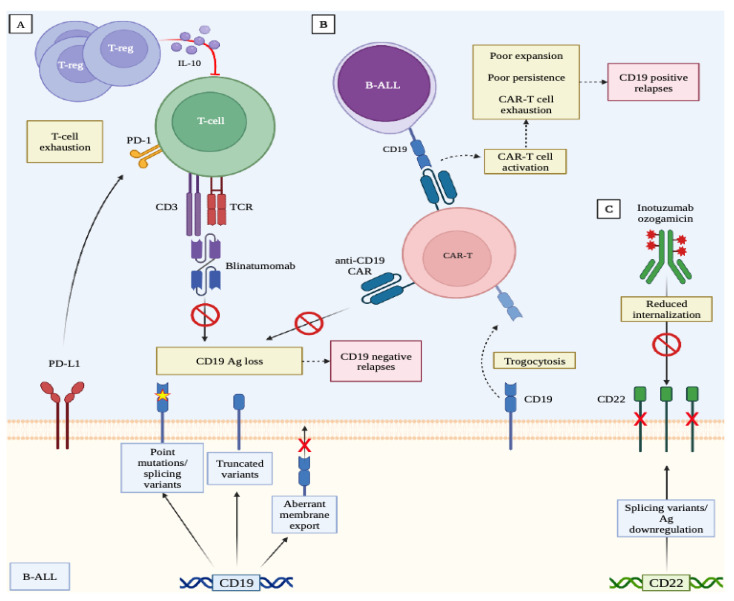
Mechanisms of relapse after exposure to different immunotherapies. Panel (**A**) T-cell activation and expansion can be impaired by the leukemia-derived mechanisms of immune escape, primarily antigen loss or phenomena derived from T-cell proliferation under chronic antigen stimulation, such as T-cell exhaustion. Panel (**B**) relapses after CAR-T cell infusion could be related to antigen loss (CD19-relapses) or to impaired CAR-T cell function (CD19+ relapses). Panel C: resistance to inotuzumab ozogamicin is mainly related to reduced CD22 expression and impaired drug internalization.

The possible mechanisms of tumor escape after exposure to the novel immunotherapies are schematized in Figure 2.

Ag represents antigen, ALL represents acute lymphoblastic leukemia, CAR represents chimeric antigen receptor, PD represents programmed death, TCR represents T-cell receptor.

The figure was created with BioRender.com.

## 4. Optimal Use of the Different Immunotherapies According to Specific Clinical Conditions

### 4.1. Extramedullary Relapse

According to the available literature, among pediatric and adult patients, nearly 40% of relapses occur in the extramedullary sites (EMD, or extramedullary disease) either isolated or along with a hematologic relapse. In the absence of head-to-head comparisons, the previously described immunotherapies seem to perform differently in this setting. In a subanalysis of the TOWER trial, blinatumomab proved to be less effective in patients with EMD [4]. Aldoss and coauthors [71] reported a lower probability of obtaining CR in patients with active EMD at the time of initiating blinatumomab or with a history of prior EMD. The same authors in a following casuistry of 132 patients confirmed similar findings: among the patients who were refractory to blinatumomab, 43% of the patients had EMD, 40% of whom had CNS involvement. In addition, for the patients who primarily responded to blinatumomab but had a history of EMD, the risk for relapse/progression at the extramedullary sites (particularly CNS) was significantly higher and not abrogated through consolidation with alloHSCT [72]. However, in a European real-life analysis on the use of blinatumomab in adult ALL patients, 10 out of 20 patients with EMD achieved complete hematological remission with 5 of them also achieving an MRD response, suggesting that the exact role of blinatumomab in this setting is not fully established [11].

In a post hoc study on the pivotal trial INOVATE, De Angelo and colleagues analyzed the efficacy of inotuzumab among 18 patients with EMD or lymphoblastic lymphoma. The CR/CRi rate was 66.7%, with 58% of the responders achieving MRD negativity. Among the patients with baseline EMD, 71% achieved CR/CRi with a durable resolution of the EMD [15].

**Figure 3:**
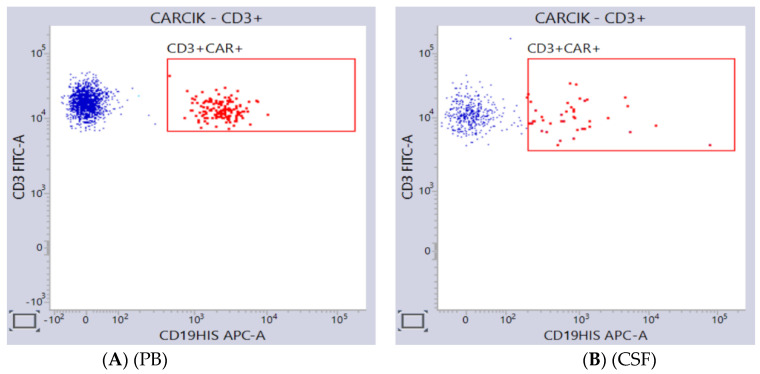
Detection of CARCIK-CD19 cells in the peripheral blood (Panel **A**, PB) and cerebral spinal fluid (Panel **B**, CSF) in an adult ALL patient. At day 28 from infusion, CARCIK-CD19 cells were simultaneously in PB (11/mcl) and CSF (1/mcl).

In a real-world analysis, the use of CAR-T cells in patients with non-CNS-EMD proved effective even though the bone marrow response was higher and quicker compared to that of the extramedullary sites [73]. Interestingly, tisagenlecleucel was safe and efficacious in a small study of 12 relapsed patients with primary CNS lymphoma. A total of 7 of the 12 patients (58.3%) obtained a response, and it was complete in 6 patients. CAR-T cells were also noted to expand and traffic to the CNS in the absence of a systemic disease [74]. Very recently, a study including 48 patients with R/R BCP-ALL with CNS involvement confirmed that CAR-T cells could induce similar high response rates in both BM and CNS diseases [75]. In keeping with these results, Shah NN et al., in a phase I study, demonstrated the efficacy of CD19-CAR-T cells in the treatment of active CNS leukemia among 13 patients with CNS involvement at infusion of which one had extensive leptomeningeal involvement [76]. In our experience with CARCIK-CD19 cells, we confirmed that these cells can move into the cerebrospinal fluid and exert antileukemic activity with manageable toxicity if the leukemic burden in this site is not overwhelming (Figure 3).

Based on the limited experience in the previously quoted studies, at our institution we considered inotuzumab to be the first option for R/R ALL patients with extramedullary disease. For patients with limited SNC involvement, CAR-T cells may represent a valuable option.

### 4.2. High-Burden Disease at Relapse

In both the pivotal trials assessing the value of blinatumomab and inotuzumab, high-burden disease was defined by a marrow blast count greater than 50%. The use of blinatumomab in this setting was clearly associated with a reduced probability of achieving CR [4,77]. Even in the setting of MRD, the disease burden matters when blinatumomab is employed. In the recent retrospective analysis of the GRAAL group, the 3-year OS of patients with molecular MRD < 0.1%, 0.1–1%, or >1% was 86%, 58%, and 33%, respectively [78]. These results suggest that an optimal ratio between the CD3+ T cells and the target CD19+ cells is probably a crucial parameter. For this reason, pre-blinatumomab mild cytoreduction should be considered whenever possible to improve leukemic control and to reduce the risk of cytokine release syndrome (CRS) and neurologic toxicity (ICANS) [52,53,79].

A post hoc analysis of the INOVATE study [15] divided patients into three groups according to their baseline blast counts: low (< 50%), moderate (50–90%), and high (>90%). The authors found that inotuzumab’s activity is independent from the disease burden; moreover, the rate of MRD negativity among the responders did not seem to be affected by the baseline disease burden. In contrast to blinatumomab, a significant clinical benefit was also observed in the patients with a high disease burden.

Similar to blinatumomab, the impact of the disease burden in CAR-T cell therapy seems to be unfavorable. In a study from MSKCC [27], patients with a higher disease burden (BM > 5% blasts) showed a lower CR rate. A recent work on a large pediatric population analyzing the impact of blinatumomab and subsequent anti-CD19 CAR-T cell therapy showed that a nonresponse to blinatumomab and a high burden disease (>5% blasts in the bone marrow) were associated with the worst EFS and RFS independent of other prognostic factors [80].

In summary, these data suggest a preference for inotuzumab for patients with high-burden disease. In such cases, a proper tumor lysis syndrome prophylaxis and, possibly, inpatient management for the first cycle could be considered. The use of blinatumomab likely remains preferable in patients lacking a robust expression of CD22, those who were previously treated with inotuzumab, or those with a higher risk of hepatotoxicity. In this respect, no more than two cycles of inotuzumab should be advised in patients who are candidates for subsequent consolidation with allogeneic transplantation. Inotuzumab may also represent an ideal bridging therapy for CAR-T cells due to the possibility of providing a sequential target therapy on two different antigens.

### 4.3. Blinatumomab, Inotuzumab, and CAR-T Cells: Do They Stand Alone or Bridge Treatments to Allogeneic Transplants?

At present, the need for subsequent alloHSCT in patients successfully treated with blinatumomab, inotuzumab, or CAR-T cells remains a matter of debate. This uncertainty is partly due to the fact that none of the published studies were designed to assess the role of alloHSCT in posttreatment consolidation, and the decision for alloHSCT was left to the treating physician. With these limitations, a clear clinical benefit for patients proceeding to alloHSCT after blinatumomab has not yet been demonstrated [3,81] but has been repeatedly suggested [11,82]. In particular, blinatumomab maintenance therapy (≥ six cycles) was associated with an increased OS and RFS compared with patients not receiving maintenance independently from consolidation with alloHSCT [83]. In the MRD setting, the OS was not different in patients who did or did not undergo transplantation, whereas, for those treated during their second or later CR, the outcome of patients who did not undergo transplantation was inferior [10]. Nonetheless, in a long-term analysis of a phase II study conducted in the MRD setting, younger patients (≤ 35 years) who underwent alloHSCT had a better 3-year survival compared with those who did not (62% vs. 22%) [82]. Finally, a single-center experience confirmed alloHSCT after blinatumomab salvage therapy to be an effective postremission treatment, with a 2-year RFS of 40% [84].

A post hoc analysis of the INOVATE trial revealed better outcomes in the patients achieving MRD negativity and proceeding to alloHSCT compared to the nontransplanted subjects [85,86]. In a pooled analysis of the phase I/II and phase III studies, the best outcome was observed in the patients who underwent alloHSCT directly upon first remission [87]. The median OS was not reached with a 2-year survival probability of 51%. As expected, the MRD negative patients had a better median posttransplant OS and PFS compared to the MRD positive patients (17.8 months vs. 5 months and 10.8 months vs. 3.4 months, respectively).

When considering CAR-T cells, different studies showed high rates of response and a prolonged survival with a different proportion of patients subsequently treated with alloHSCT after treatment with CAR-T cells. The pivotal phase II trial ELIANA showed a rate of RFS up to 60% at 1 year and 49% at 5 years, with only a minority of patients receiving alloHSCT [24,25]. Using a different CAR-T construct, Lee et al. demonstrated a role of transplantation in consolidation in reducing the relapse rate in young patients with R/R BCP-ALL [88]. Similarly, in a phase I trial using CD19.28 CAR-T cells in R/R children and AYAs, the patients with a molecular response who received a transplant showed a sustained response and survival at long-term follow-up. All the MRD negative patients not proceeding to alloHSCT relapsed at a median time of 5 months post-CAR-T cell infusion [76]. In the adult setting, conflicting results have been reported on the role of consolidative alloHSCT in adult patients achieving MRD negative remission after CAR-T cell therapy. Park and colleagues showed no differences in the survival outcomes between the patients receiving alloHSCT after achieving MRD negativity compared to those who did not [27]. Similarly, when analyzing the recent results of the phase II ZUMA-3 trial, Shah and colleagues showed no effect of alloHSCT on the duration of response nor on the RFS. However, the proportion of the patients receiving transplantation was significantly low (18%) [29]. In contrast, three other studies showed that alloHSCT in adult patients achieving MRD negativity after CD19 CAR-T cell therapy was associated with an improvement in the EFS. However, this benefit did not translate into a clear benefit in terms of the OS [31,32,33].

All in all, with the novel immunotherapies, a prolonged clinical benefit can be achieved in a fair proportion of the responding patients who also achieved an MRD negative status. In this context, a subsequent alloHSCT should be taken into consideration as a possible effective strategy at least for younger and fit patients in order to limit the risk of an early relapse after immunotherapy.

Table 2 summarizes our suggested approach according to different clinical conditions.

## 5. Future Perspectives

### 5.1. Anti-CD19 and Anti-CD22 Immunotherapies as Frontline Treatments

Both anti-CD19 and anti-CD22 immunotherapies have been explored in recent clinical trials as frontline treatments in association or sequentially to chemotherapy. In the phase II study GIMEMA LAL2317 (NCT03367299), blinatumomab was added to a pediatric-like risk-oriented regimen in a sequential manner following the early and late consolidation phases based on high-dose methotrexate and ara-C. Upfront alloHSCT was limited to patients with a very high clinical or cytogenetic risk profile at diagnosis and patients remaining MRD positive (i.e., ≥ 10^−4^) after early consolidation. A total of 149 BCP-ALL Ph-negative patients were enrolled (median age of 41 and range of 18–65) of whom 90% obtained a complete hematological response. No matter the MRD status after consolidation, blinatumomab was given to all the patients. MRD negativity improved from 72% achieved after consolidation chemotherapy up to 95% following one blinatumomab cycle (*p* = 0.018) [89]. The use of blinatumomab in first-line treatment was also recently explored in a phase II single-arm monocentric study by the MD Anderson researchers using a sequential approach of four cycles (instead of eight) of high-dose induction chemotherapy based on the hyper-CVAD/MTX-ara-C scheme followed by four cycles of blinatumomab. The immunotherapy was also incorporated in the maintenance scheme and was alternated with POMP chemotherapy. MRD was detected through flow cytometry (MFC) and through next-generation sequencing. In the updated results of this trial, with a median follow-up of 37 months, all 32 of the untreated patients obtained a complete response at any time during treatment, with 97% of the subjects achieving MRD negativity through MFC overall. Of note, 12% of the patients that received blinatumomab converted their MRD positivity after the first cycle of treatment. Treatment was well tolerated, with less than half of the patients experiencing a neurological toxicity of any grade, and there was only one discontinuation due to treatment-related neurotoxicity [90]. Although with the limit of a short follow-up, the improved MRD responses in the above-mentioned studies were also associated with superior survival outcomes compared to historic controls. With a median follow up of 10 months, the GIMEMA trial reported a 1-year OS and DFS of 84% and 72%, respectively [89]. Likewise, the patients treated in the MD Anderson study experienced an estimated 3-year PFS and OS of 73% and 81%, respectively [90]. Other cooperative groups are favorably applying these immunotherapies as frontline therapies in ongoing clinical studies. The Eastern Cooperative Oncology Group (ECOG) recently completed and presented at the 64th American Society of Hematology Annual Meeting a phase III trial comparing standard chemotherapy with or without blinatumomab (NCT02003222). In this study, the patients with MRD negativity at the end of induction were randomized to receive a standard course of consolidation chemotherapy with or without blinatumomab. The results showed that patients receiving blinatumomab had an OS of 83% compared with 65% in the control arm [91]. Furthermore, the Dutch–Belgian phase II HOVON146ALL trial is now evaluating the ability of blinatumomab in addition to prephase treatment and consolidation therapy to induce a deeper molecular response and to possibly obtain durable remission (NCT03541083).

Taking into consideration the good safety profile shown by inotuzumab in combination with chemotherapy in the relapse setting, this association is now being explored in frontline treatments. In particular, a phase III trial comparing inotuzumab in addition to standard induction treatment in newly diagnosed young adult BCP-ALL patients is ongoing (NCT03150693). This study could further elucidate the feasibility of inotuzumab as a first-line treatment with intense chemotherapy in terms of the response rate, survival advantage, and expected toxicity. A summary of the main studies exploring the immunotherapies is available in the Supplementary Materials (Table S1).

### 5.2. Should Less Intensive Chemotherapy or a Chemo-Free Approach Be Used to Treat Old and Frail Patients?

The treatment landscape of older patients is rapidly changing, and their results are improving thanks to the addition of innovative immunotherapies. The dismal outcome of older patients with an expected 5-year overall survival of less than 20% [92] will rapidly improve thanks to these innovative treatments. The MD Anderson group proposed a reduced-intensity chemotherapy based on eight cycles of mini-hyper-CVD associated with fractionated inotuzumab for the first four cycles for newly diagnosed patients aged ≥ 60 years. The study protocol was further amended to incorporate four cycles of blinatumomab as a consolidation treatment, reducing the chemotherapy to four cycles and lowering the dose of fractionated inotuzumab. Blinatumomab was continued as a maintenance treatment alternated with POMP. Among 64 patients evaluable for a morphologic response with a median age of 68 years (range of 60–81), 96% achieved an MRD negativity overall. The induction mortality was very low (< 5%), and the incidence of VOD was < 10% [93]. Interestingly, a recent propensity score analysis on 58 old patients treated with mini-HCVD and inotuzumab revealed improved complete responses and outcomes when compared to historic controls [19]. The German cooperative group GMALL evaluated the efficacy of inotuzumab as an induction treatment followed by a conventional high-dose consolidation chemotherapy. Patients aged ≥ 55 years were enrolled in this study. Complete remission was achieved by all the 27 patients who completed the induction phase, with 74% of the patients available for the MRD study reaching a molecular response. Reported severe adverse events were mainly hematological, while approximately one-third of the patients experienced a grade ≥ III transaminitis. No VOD cases were reported [94].

The role of blinatumomab as the first-line treatment for older patients with BCP-ALL was investigated in the SWOG 1318 trial. In this chemo-free protocol, the bispecific antibody was given as an induction treatment for two cycles followed by an additional three consolidation cycles and POMP maintenance. Treatment was well tolerated, with no early deaths reported during the induction phase. Of the 29 evaluable patients (median age of 75 years), two-thirds obtained a complete response, with an estimated 3-year DFS and OS of 37% and 37%, respectively [95]. In line with previous studies, two phase II clinical trials performed by the European working group on adult ALL (EWALL) are evaluating the combination of either inotuzumab (NCT03249870) or blinatumomab (NCT03480438) with mild-intensity chemotherapy in patients older than 55 years (see the Supplementary Table S1).

### 5.3. The Use of Immunotherapies in Ph-Positive ALL

In the Ph-positive setting, the introduction of tyrosine kinase inhibitors (TKIs) radically changed the natural history of this otherwise poor-prognosis subset. Given the high prevalence of older patients in this subset of BCP-ALL patients, several studies investigated the association of different TKIs with less-intensive chemotherapy, showing a high rate of induction responses and a lower induction toxic mortality, although the best consolidation strategy is still a matter of debate [92]. In this regard, several clinical trials are evaluating the best consolidation strategy with a particular interest in chemo-free schemes based on the association of TKIs and the novel immunotherapies.

Following pioneering studies showing a high remission rate with TKIs only [96,97,98], the GIMEMA phase II trial D-ALBA investigated a chemo-free program based on the sequential administration of dasatinib and blinatumomab. Of the 63 enrolled patients (median age of 54 years), all but 1 obtained a complete response after the induction treatment. Interestingly, the rate of the molecular response obtained after the first two cycles of consolidation therapy further improved with subsequent blinatumomab infusions, with more than 80% of patients being MRD negative after four cycles of blinatumomab consolidation [99]. Based on the excellent results of D-ALBA, the GIMEMA group is now heading a phase III randomized clinical trial comparing the combination of third-generation TKI ponatinib and blinatumomab with the association of imatinib and chemotherapy (GIMEMA ALL2820 trial, NCT04722848). The MD Anderson group very recently published the results of a phase II study based on the combination of ponatinib and blinatumomab in newly diagnosed or R/R Ph-positive BCP-ALL patients. The bispecific antibody was given up to five cycles, while TKI monotherapy was continued as a maintenance treatment for at least five years. Among the patients in the first-line treatment (40 patients, median age of 57 years), they all achieved a complete response of whom 33 of 38 evaluable patients (87%) had a molecular response. At 16 months of follow-up, the 1-year EFS and OS were both 95%; one patient died in CR due to hypovolemic shock following cardiac catheterization, and one patient received alloHSCT due to a persistently detectable BCR-ABL1 transcript [100].

The recent and ongoing clinical trials on frontline immunotherapy in Ph+ ALL are available in the Supplementary Table S1.

### 5.4. Combination with Checkpoint Inhibitors

Among patients who relapsed after the immunotherapies, different mechanisms of escape have been described, and new studies are now investigating the efficacy of new combinations of treatments that target those pathways. In the patients who relapsed after blinatumomab, there is evidence that leukemic cells and the tumor microenvironment can lead to T-cell suppression. In particular, PD-L1 overexpression on BCP-ALL blasts and the upregulation of T-reg cells have been described as possible phenomena of resistance in relapsed patients [51]. Thus, the possible options of treatment are the combination of blinatumomab with checkpoint inhibitors in CD19-positive relapses [49,101,102]; the use of CAR-T cells with a different affinity for CD19 [103]; the use of other immunotherapies, such as inotuzumab, in CD19-negative cases, or the use of bicistronic CAR-T cells targeting CD19 and CD22 [104,105]. Many clinical trials combining blinatumomab and checkpoint inhibitors or new CAR-T cell constructs are currently ongoing (Table S2 in the Supplementary Materials).

## 6. Conclusions

Based on the results obtained in the R/R setting showing the ability of immunotherapies to induce not only hematologic but also molecular remission, several trials are currently evaluating their use as part of frontline treatments. All these trials may lead to the development of an innovative combination of chemoimmunotherapies for patients with ALL with less toxicity and to the establishment of their best positioning in future upfront therapeutic regimens with the goal of increasing the rate and quality of deep molecular responses in ALL (Figure 1A,B and Table S1 in the Supplementary Materials). In our opinion, blinatumomab or inotuzumab could be better incorporated into the earlier phases of treatment based on some characteristics, such as their availability off the shelf and no need of lymphodepleting chemotherapy, leaving a wider therapeutic role of CAR-T cells in an earlier R/R setting. The encouraging preliminary results coming from the ongoing studies suggest that immunotherapies really have the potential to reduce the need to use intensive chemotherapy or to reduce the indication of alloHSCT as a consolidation treatment. In this regard, the combination of blinatumomab and the new generation of TKIs in Ph+ ALL represents the first evidence that a frontline chemotherapy-free regimen can be very effective, also avoiding the need for alloHSCT in most patients. Thus, the landscape of treatment is now really changing, and, in the coming years, a wider use of these drugs in frontline treatments is likely. The clinical benefit of incorporating these drugs will not be limited to younger and fit patients but will also apply to older and more frail patients when considering that the immunotherapies are better tolerated than conventional chemotherapy. At the same time, these immunotherapies could improve transplantation outcomes when given either as a prophylaxis or as a postrelapse treatment [106].

## Figures and Tables

**Table 1:** Summary of the main clinical studies on the immunotherapies approved for treating R/R BCP-ALL (blinatumomab, inotuzumab, tisagenlecleucel, and brexucabtagene autoleucel).

Trial/Study [Reference]	Setting	Study Design	Total Patients	Immunotherapeutic Treatment	Main Results
Tower 2017 [13]	R/R Ph-negative adults	Phase III randomized trial	271 blinatumomab vs. 134 ChT	Blinatumomab 9 µg/day first week then 28 µg/day c.i.	CR: blinatumomab 34% vs. ChT 16%. Molecular response: blinatumomab 76% vs. ChT 48%. Median RFS: blinatumomab 7.3 vs. ChT 4.6 months. OS: blinatumomab 7.7 vs. ChT 4.0 months.
AALL1331 [6]	R/R Ph-negative children and young adults (1–30 years)	Phase III randomized trial	107 blinatumomab vs. 109 ChT	Blinatumomab 15 μg/m^2^ day c.i. for 28 days	Molecular response: blinatumomab 75% vs. 32% ChT. RFS: blinatumomab 54% vs. ChT 39%. OS: blinatumomab 71% vs. ChT 58%.
Locatelli et al. [7]	High risk first relapse Ph-negative children	Phase III randomized trial	54 blinatumomab vs. 54 ChT for the 3rd consolidation before alloHSCT	Blinatumomab 15 μg/m^2^ day c.i. for 28 days	Minimal residual disease remission: blinatumomab 90% vs. ChT 54%. EFS: blinatumomab 66% vs. ChT 27%. OS: blinatumomab 85% vs. ChT 70%.
ALCANTARA [8]	R/R Ph-positive adults	Phase II trial	45	Blinatumomab 9 µg/day first week then 28 µg/day c.i.	CR: 36%. Molecular response: 88%. Median RFS: 6.7 months. OS: 7.1 months.
Blast [10]	MRD positive after induction	Phase II trial	113	Blinatumomab 15 µg/m^2^/day c.i.	Molecular response: 78%. Medina RFS: 18.9 months. OS: 36.5 months.
INO-VATE ALL [14]	R/R Ph-negative or -positive adults	Phase III randomized trial	109 inotuzumab vs. 109 ChT	Inotuzumab 0.8 mg/m^2^ on day 1 of each cycle and 0.5 mg/m^2^ on days 8 and 15	CR: inotuzumab 81% vs. ChT 29%. Molecular response: inotuzumab 78% vs. ChT 28%. Median RFS: inotuzumab 5 months vs. ChT 1.8 months. OS: inotuzumab 7.7 vs. ChT 6.7 months. VOD: inotuzumab 13% vs. ChT 1% (most cases occurred after alloHSCT).
AALL1621 [21]	R/R Ph- and Ph+ children and adolescents (age of 1–21 years)	Phase II trial	48	Inotuzumab dosing was 0.8 mg/m^2^ intravenously on day 1 and 0.5 mg/m^2^ on days 8 and 15 of a 28-day cycle	CR: 58%. MRD response: 67%. Minimal residual disease measured by flow cytometry: 18 (66.7%) had minimal residual disease < 0.01%. VOD: 29% among 21 patients undergoing alloHSCT. Partial CD22 expression and lower CD22 site density were associated with a lower likelihood of response to inotuzumab.
Eliana study [23,24,25]	R/R Ph- and Ph+ children and young adults (age of 3–21)	Phase II trial	79	Single infusion of tisagenlecleucel	CR rate: 82%. MRD negativity: 100%. 5-year RFS: 49%. 5y OS: 55%.
Tisagenlecleucel real-world study [28]	R/R Ph- and Ph+ children and young adults (age of 1–26)	Real-world setting	255	Single infusion of commercial tisagenlecleucel	CR: 86%. DOR: 61%. EFS: 52%. OS: 77%.
ZUMA-3 trial [29]	R/R Ph- and Ph+ adults	Phase II trial	55	Single infusion of brexucabtagene autoleucel	CR: 71%. MRD response: 97%. Median DOR: 13 months. Median RFS: 12 months. Median OS: 18 months.

R/R represents relapsed/refractory, Ph represents Philadelphia, ChT represents chemotherapy, c.i. represents continuous infusion, CR represents complete remission, RFS represents relapse-free survival, OS represents overall survival, EFS represents event-free survival, MRD represents measurable residual disease, VOD represents veno-occlusive disease, and alloHSCT represents allogeneic stem cell transplantation.

**Table 2:** Summary of suggested strategies according to different clinical conditions.

Condition	Our Approach
Extramedullary relapse	Inotuzumab or CAR-T cells as the first option. In patients lacking CD22 surface expression or those previously treated with inotuzumab, blinatumomab is a valid alternative. For patients with SNC involvement (if not rapidly progressive), CAR-T cells may represent a valuable option.
High-burden disease at relapse	Inotuzumab should be preferred if bone marrow blasts are >50%. For patients with CD22 negativity, those previously treated with inotuzumab, or those at high risk of hepatotoxicity, blinatumomab is a valid option. In this case, mild cytoreductive therapy before starting blinatumomab is advised.
Consolidation treatment after CR achievement with or without alloHSCT	In younger and fit patients achieving an MRD negative CR after inotuzumab or blinatumomab, we usually advise further consolidation with their first alloHSCT. For those relapsed after a previous transplant, a second alloHSCT should be discussed with the patient. MRD negative responders after CAR-T cell therapy may proceed to their subsequent first alloHSCT, and this option should be discussed with the patient. In younger and fit patients with an MRD positive hematologic remission, alloHSCT is always advised. In older or frail patients not eligible to receive alloHSCT, additional continuation/maintenance therapy with blinatumomab or inotuzumab should be considered. Sequential treatments with inotuzumab, blinatumomab and/or CAR-T cells are potentially valid and should be considered in the setting of clinical studies (some are already underway).

## Supplementary Materials

The following supporting information can be downloaded at https://www.mdpi.com/article/10.3390/cancers15041349/s1, Table S1: Recent and ongoing studies exploring immunotherapies in the frontline treatment of BCP-ALL patients and Table S2: Novel approaches in relapse/refractory BCP-ALL patients.
