# Supplementary material for: Optimal Use of Novel Immunotherapeutics in B-Cell Precursor ALL
Source: Cancers (Basel). 2023 Feb 20;15(4):1349. doi: 10.3390/cancers15041349 (PMC9954469; doi:10.3390/cancers15041349)
Supplement: Supplementary file 1 [file cancers-15-01349-s001.zip › cancers-2177979-supplementary.pdf]

Table S1. Recent and ongoing studies exploring immunotherapies in the frontline treatment of B-ALL patients

Trial number	Cooperative Group	Phase	Planned N of pts	Age	Treatment	Position of the immunotherapy	Ref./DOI
**B-ALL Ph negative**							
NCT02877303 (NCI-2017-00596)	M.D. Anderson	Phase II	80	14-59	ChT plus blinatumomab (+/- inotuzumab)	> Blinatumomab plus InO (day 5, 11 on even cycles) for up to 4 cycles after induction ChT;<br>> Maintenance: Blinatumomab alternating with ChT	[107]
NCT03150693 (Alliance A041501)	Alliance (Dana-Farber)	Phase III	310	18-39	Frontline ChT (Arm 1) vs<br>Frontline ChT plus inotuzumab (Arm 2)	Inotuzumab for two cycles after remission induction treatment, before consolidation ChT	
NCT03367299 (GIEMEMA LAL2317)	GIMEMA	Phase II	149	18-65	ChT plus blinatumomab	Blinatumomab after high-dose consolidation cycles (cycle 3 and 6)	[89]
NCT03541083 (HOVON146ALL)	HOVON	Phase II	80	18-70	Blinatumomab plus ChT	> Prephase: blinatumomab plus steroid;<br>> Blinatumomab after consolidation and after late intensification therapy, before maintenance or alloHSCT.	
NCT02003222 (ECOG-E1910)	ECOG-ACRIN	Phase III	488	30-70	Frontline ChT vs Frontline ChT plus blinatumomab	> Blinatumomab after first intensification cycle and after Cycle 3 of consolidation;<br>> Blinatumomab before maintenance treatment	[91]

NCT03249870 (EWALL-INO)	EWALL	Phase II	130	>55	Fractionated inotuzumab plus dose-reduced ChT	> Induction part I: reduced-intensity ChT plus 3 injections of inotuzumab (0.8 mg/mq day 1; 0.5 mg/mq day 8 and 15)<br>> Induction part II (pts in CR/CRp or salvage treatment): ChT plus 2 injections of InO 0.5 mg/mq (day 1, 15)	[108]
NCT03480438 (EWALL-BOLD)	EWALL-GMALL	Phase II	50	56-74	Blinatumomab plus dose-reduced ChT	> induction: 1 cycle of blinatumomab after ChT. Same scheme is repeated in patients failing Induction I.<br>> Consolidation: Cht alternating with Blinatumomab for a total of 6 cycles (3 ChT and 3 infusions of blinatumomab)	EHA Library. Gökbuget N. 06/12/2020; 294333; EP414
NCT03460522 (INITIAL-1)	GMALL	Phase II	45	56-74	Inotuzumab plus ChT	Inotuzumab 1.8 mg as induction, followed by consolidation with 2 cycles with InO 1.5 mg plus conventional ChT	[94]
NCT01371630 (NCI-2011-01123)	M.D. Anderson	Phase I/II	276	≥60	Inotuzumab (+/- blinatumomab) plus mini-Hyper-CVD	> Induction: reduced-intensity ChT followed by fractionated inotuzumab for four cycles,<br>> Consolidation: 4 cycles of Blinatumomab<br>> Maintenance: blinatumomab alternating with ChT	[109]
NCT03739814 (Alliance A041703)	Alliance	Phase II	64	≥60	Inotuzumab plus Blinatumomab	> Induction: inotuzumab (21-day course) up to 2 cycles<br>> Consolidation: blinatumomab (84-days course) up to 2 cycles	
NCT02143414 (SWOG 1318)	SWOG	Phase II	58	> 65	Blinatumomab plus maintenance	> Induction: blinatumomab up to 2 cycles.<br>> Consolidation: blinatumomab for 3 cycles	[95]

**B-ALL Ph positive**							
NCT02744768 (D-ALBA)	GIMEMA	Phase II	60	≥18	Dasatinib plus blinatumomab	blinatumomab plus TKI as consolidation for up to 5 cycles, after a 84-day induction with TKI plus steroid	[99]
NCT04722848 (GIEMEMA ALL2820)	GIMEMA	Phase III	236	≥18	Ponatinib plus blinatumomab<br>vs<br>Imatinib plus CT	Consolidation with blinatumomab up to 5 cycles after a 70-day chemo-free induction with TKI plus steroid	
NCT04530565 (EA9181)	ECOG-ACRIN	Phase III	330	18-75	TKI (dasatinib or ponatinib) plus ChT<br>vs<br>TKI plus blinatumomab	Blinatumomab is associated with TKI for 2 cycles in a chemo-free induction treatment after a short pre-phase with steroid and TKI.	
NCT03147612 (NCI-2018-01186)	M.D. Anderson	Phase II	60	>18	Ponatinib plus blinatumomab and low-intensity ChT	Blinatumomab for 4 cycles as consolidation treatments after an induction treatment based on TKI plus reduced-intensity ChT.	
NCT03263572 (NCI-2018-01078)	M.D. Anderson	Phase II	60	>18	Ponatinib plus blinatumomab	Blinatumomab is administered as induction therapy up to 5 cycles in association with TKI. TKI is continued for at least 5 years.	[110]
NCT04688983 (EWALL-Ph-03)	EWALL	Phase II	180	≥55	Arm1: ponatinib plus standard ChT<br>Arm2: imatinib plus standard ChT<br>Arm3: ponatinib plus blinatumomab	In the experimental arm, TKI is given continuously in association with blinatumomab.	

ChT: chemotherapy; TKI: tyrosine kinase inhibitor

Table S2. Novel approaches in relapse/refractory B-ALL patients

Trial number	Cooperative Group	Phase	Planned N of pts	Age	Treatment	Study overview	Ref.
**Check-point inhibitors**							
NCT04546399 (AALL1821)	NCI	II	550	≤30	Blinatumomab plus nivolumab	A Pre-phase treatment is given to patients with EM relapses or hyperleukocytosis, and re-induction is dispensed to young patients or those with late or EM relapses. Patients are then randomized to receive blinatumomab with or without nivolumab (on day 11, 25 of C1; on day 1, 15 of C2) up to two cycles.	
NCT03160079 (161287/UCHMC1504)	Single center (UCHMC)	I/II	24	≥18	Blinatumomab plus pembrolizumab	Blinatumomab is administered in association with Pembrolizumab i.v. given on day 15 and 36 of a 42-day cycles for two cycles. Then, patients in CR/CRh continue for a total of 5 cycles.	[102]
NCT03512405 (NCI-2018-00526)	Single center (City of Hope)	I/II	36	≥18	Blinatumomab plus pembrolizumab	Patients receive blinatumomab plus pembrolizumab infused on day 15 or 22 in cycle 1, then on day 1 and 22 of a 42-day cycle. Up to 5 cycles are permitted. Patients in CR could receive a maintenance treatment with Blinatumomab for up to 4 cycles.	[111]
NCT02879695 (NCI-2016-01300)	NCI	I	30	≥16	Blinatumomab plus nivolumab or blinatumomab plus nivolumab and ipilimumab	Blinatumomab is given in association with nivolumab i.v. on day 11 and then every two weeks of a 42-cycle for one year. In a group of patients, Ipilimumab is added in day 11 every 6 weeks for one year.	[101]

**CAR constructs**							
NCT03241940	Academic, Lucile Packard Children's Hospital, Stanford University	I	50	≤30	Autologous CD19/CD22 CAR-T	Dose escalation study of a dual antigen CD19/CD22-CAR-T cell product. Cells are infused after lymphodepletion with Flu-CY. Four different cell doses are explored.	
NCT03330691 (PLAT-05)	Academic, Seattle Children's Hospital	I	80	≤30	SCRI-CAR19x22v1 and SCRI-CAR19x22v2 (Autologous CD19/CD22 CAR-T)		[112]
NCT03448393 (18-C-0059)	NCI	I	87	3-39	CD19.22.BBz (Autologous CD19/CD22 CAR-T)	Transduced autologous T cells express a bivalent CD19/CD22 CAR. In order to optimize CAR-T cell expansion and persistence, a bicistronic CD19/CD22 CAR construct is further explored.	[113]
NCT04029038 (NCI-2019-04229)	M.D. Anderson	I/II	30	≤70	Autologous CD19/CD22 CAR-T	Patients receive a standard lymphodepleting therapy based on Flu-CY followed by the infusion of CD19-CD22 CAR-T cell product. Patients relapsing after the protocol assessment could receive a second infusion of engineered cells.	
NCT03289455 (AMELIA)	Industry	I/II	23	<25	AUTO3 (CD19/22 CAR-T)	AUTO3 is an autologous CAR-T cell product expressing humanized bicistronic anti-CD19/CD22 CAR. The CAR construct incorporates the TNFR costimulatory domain.	[114]

NCT03620058	Academic, University of Pennsylvania	I	23	≥18	CART22-65s plus huCART19	CART22-65s and huCART19 are two humanized autologous CAR-T cell products explored in patients with R/R B-ALL. Two million cells/kg per cell product are infused after Flu-CY lymphodepletion.	[115]
NCT03825718	Academic, Hebei Yanda Ludaopei Hospital	I	25	3-44	CD19 Fast-CAR-T (F-CAR-T)	CD19 F-CAR-T are cells frozen few hours after the viral transduction to allow the CAR-T cell expansion in vivo after infusion back into the patient. Reducing the duration of ex vivo culture should limit the differentiation of T cells, favoring the preservation of naïve and stem cell memory T cells in the final product	[36]
NCT02746952 (CALM)	Industry	I	25	16-69	UCART19 (allogeneic engineered anti-19 CAR-T)	Donor-derived allogeneic anti-CD19 CAR-T cells are engineered with TALENs nucleases disrupting TCR alpha chain (TRAC) and CD52 genes, reducing the risk of GvHD and acquiring resistance to lymphodepleting drugs. The product is infused after a lymphodepleting regimen containing Flu-CY and alemtuzumab.	[40]
NCT04150497 (BALLI-01)	Industry	I	30	15-70	UCART22 (allogeneic engineered anti-22 CAR-T)	Allogeneic lymphocytes are transduced with lentivector expressing anti-22 CAR construct. TRAC and CD52 genes are disrupted using TALENs. In order to increase CAR-T expansion, alemtuzumab is added to lymphodepletion with Flu-CY.	[116]
NCT03666000 (PBCAR0191-01)	Industry	I/II	120	≥18	PBCAR0191 (allogeneic engineered anti-19 CAR-T)	The CD-19 CAR is inserted in the TRAC locus of donor-derived T cells using a TRAC-specific ARCUS nuclease, disrupting the endogenous TCR and preventing GvHD. A standard (SL) or enhanced lymphodepleting chemotherapy	[117]

						(i.e. Flu 30 mg x4 days and Cy 1000 mg x3 days) is investigated.	
NCT03389035 -(FT01CARCIK)	Academic Fondazione MBBM Monza and ASST Papa Giovanni XXIII Bergamo	I/II	21	1-75	CARCIK-CD19 (anti-CD19 Cytokine-induced killer cells)	Donor-derived, in vitro differentiated CIK cells engineered using a non-viral Sleeping Beauty transposon system which induces the expression of an anti-CD19 CAR construct. Infused in patients R/R after alloHSCT, donor-derived CARCIK-CD19 overcome the HLA barrier, potentially reducing the risk of GvHD.	[38-39]
NCT05252403 (FT03CARCIK)	Academic Fondazione MBBM Monza and ASST Papa Giovanni XXIII Bergamo	II	33	1-75	CARCIK-CD19 (anti-CD19 Cytokine-induced killer cells)	To determine the activity and the safety of a therapeutic strategy that allows a second CARCIK-CD19 cells infusion, driven by the status of disease from one month after the first infusion, in adult and pediatric patients with r/r BCPALL.	

CAR: Chimeric Antigen Receptor; EM: Extramedullary; Flu-Cy: Fludarabine-Cyclophosphamide; Flu: fludarabine; Cy: cyclophosphamide; GvHD: Graft vs Host Disease; TCR: T Cell Receptor; i.v.: intravenous
